# Quality of life among caregivers of patients with schizophrenia: a cross-cultural comparison of Chilean and French families

**DOI:** 10.1186/1471-2296-13-42

**Published:** 2012-05-28

**Authors:** Laurent Boyer, Alejandra Caqueo-Urízar, Raphaelle Richieri, Christophe Lancon, José Gutiérrez-Maldonado, Pascal Auquier

**Affiliations:** 1 Aix-Marseille University, EA 3279 Research Unit, 27 Boulevard Jean Moulin, Marseille, 13284, France; 2 Departamento de Filosofía y Psicología, Universidad de Tarapacá, 18 de Septiembre # 2222, Arica, Chile; 3 Departamento de Personalidad, Evaluación y Tratamiento Psicológico, Universidad de Barcelona, Paseo Valle de Hebrón, Barcelona, 08035, Spain

**Keywords:** Quality of life, Caregivers, Cross culture, Family support, Schizophrenia

## Abstract

**Background:**

To our knowledge, no study has examined quality of life (QoL) among caregivers of individuals with schizophrenia between a developing and a developed country. The aim of this study was to assess QoL of the caregivers of individuals with schizophrenia in two countries characterized by different social, economic and cultural conditions, namely Chile and France.

**Methods:**

Data were collected from public mental health outpatient services in Arica (Chile), and in Marseille (France). QoL was measured with the short-form health survey scale - 36 items (SF36). QoL of 41 Chilean caregivers was firstly compared with 245 French caregivers. Univariate and multivariate analyses using linear regression were then performed to determine variables potentially related to QoL scores.

**Results:**

The caregivers were primarily mothers in the two groups, but Chilean caregivers were younger, and lived more frequently with the individual with schizophrenia than French caregivers. The SF36 scores were globally low in the two groups, especially on the mental QoL scores. Chilean caregivers reported lower physical SF36 scores than French caregivers. In the multivariate analysis, being mother and Chilean caregivers were the most regular features associating to a lower QoL.

**Conclusion:**

Despite differences between Chile and France, especially in terms of quality and quantity of mental health services and economic supports, caregivers’ QoL levels remain particularly low for both countries. Future support programmes should address the specific needs of caregivers.

## Background

Schizophrenia is a disabling and severe psychiatric disorder which is a major cause of suffering for patients. Schizophrenia also affects functioning and health of family caregivers, mainly because the caregivers have assumed functions that were performed in the past by psychiatric institutions [1-5]. Caregivers supply the patient with care and support [1,2]. The impact of caregiving on caregivers’ quality of life (QoL) is substantial [3-7], especially when experiencing a significant burden [8,9], restricted roles and activities, and increased psychosomatic, anxious, or depressive symptoms [1,10]. Moreover, caregivers’ negative experience may affect their ability to care for the patients [11,12]. Research on caregivers’ QoL is thus of importance both for the caregivers themselves and indirectly for patients’ health.

Caregivers of individuals with schizophrenia have received significant attention in the past few years. Previous studies mainly documented association of burden with socio-demographic characteristics, coping and social support of caregivers, and severity of symptoms and disabilities of patients [2,5,13-16]. More rarely, social, economic and cultural factors were studied although there is some evidence that these factors have a potential influence on caregiving consequences. Cross-cultural or cross-national comparisons of caregivers would help to understand these factors, but only few studies to date have been performed, in particular in developed countries [14,15,17]. Moreover, these studies were focused on caregivers’ burden. In this area, there is an interest in studying caregivers’ QoL from different social, economic and cultural contexts.

The aim of this study was thus to assess QoL of the caregivers of individuals with schizophrenia in two countries characterized by different social, economic and cultural conditions, namely Chile and France, using the short-form health survey scale - 36 items (SF36).

## Method

### Settings

The study was conducted in public mental health outpatient services in Arica (Chile), and in Marseille (France). Arica is a city in northern Chile, with a population of slightly under 195,000 inhabitants. Marseille is located in the south-eastern part of France with a population of nearly one million inhabitants. Chile and French Heath systems are described in Additional file 1.

### Participants

The inclusion criteria for the caregiver were as follows: (1) having a family member with a diagnosis of schizophrenia according to the DSM-IV criteria [18]; (2) being identified by the individual with schizophrenia as the main caregiver; (4) being 18 years of age or older; and (5) giving informed consent to participate in the study. In Chile, the study was approved centrally by the Ethics Committee of the University of Tarapacá and subsequently by each participating mental health services. In France, the university research team received an agreement from the French Institute for Public Health Research with support from the Ministries of Health and Research to conduct QoL researches. This study was conducted in accordance with the Declaration of Helsinki and French Good Clinical Practices [19,20].

### Procedure

In Chile as in France, personnel from each public mental health outpatient service identified consecutive patients who had been given a diagnosis of schizophrenia and were between 18 and 64 years old on a period of one month. Each patient was asked by medical or nursing staff to name his or her main caregiver, i.e. the person who spends the most time supporting and taking care of the patient. We asked the patient if we could contact the caregiver. When the patient agreed and when the caregiver met the inclusion criteria, clinical psychologist explained the aim of the evaluation and invited caregivers to take part. After providing consent to participate in the study, caregivers were assessed individually in a private booth via a self-report questionnaire and an interview. In Chile, 45 caregivers of patients with schizophrenia were initially approached and 41 agreed (acceptance rate = 91%). In France, 265 caregivers were approached and 245 agreed to participate (acceptance rate = 93%).

### Data collection

The data collected included the following:

1. Socio-demographic characteristics of the caregivers: Gender, age, and employment status.

2. Socio-demographic characteristics of the individuals with schizophrenia: Gender, and age.

3. QoL questionnaire: the Short Form 36 (SF36) was used to assess QoL [21]. SF36 is a generic, self-administered, and worldwide-used questionnaire consisting of 36 items describing 8 dimensions: Physical Functioning (PF), Social Functioning (SF), Role-Physical Problems (RPP), Role-Emotional Problems (REP), Mental Health (MH), Vitality (VIT), Bodily Pain (BP), and General Health (GH). Each dimension is scored within a range of 0 (low QoL level) to 100 (high QoL level). Two component summary measures of SF36, namely physical and mental composite scores (PCS-SF36 and MCS-SF36) can be calculated. The use of SF-36 in caregivers of individuals with mental health problems was recommended in a recent review of the available instruments [22]. Moreover, the SF-36 allows comparisons between French and Chilean populations, cross-cultural validation showing that the Chilean and French versions of the SF-36 have the same subscales and structure as the original SF-36 and have demonstrated adequate psychometric properties.

### Statistical analysis

Data were expressed in proportion or mean and standard deviation. Characteristics of Chilean and French caregivers were compared using Chi-squared or Fisher exact tests for categorical variables and Student or Wilcoxon rank sum test for continuous variables.

Associations between QoL scores and continuous variables (caregiver: age; patient: age) or categorical variables (caregiver: relationship, living situation, employment status; patient: gender; country) were analyzed using Pearson’s correlation and Chi-squared tests. Multivariate analyses using linear regression were performed to determine variables potentially related to QoL scores. The final models expressed the standardised beta coefficient. All the tests were two-sided. Statistical significance was defined as p < 0.05. Statistical analysis was performed using the SPSS version 17.0 software package (SPSS Inc, Chicago, IL, USA).

## Results

### Comparisons of characteristics and SF36 scores between Chilean and French caregivers

Table 1 presents the characteristics and SF36 scores of Chilean (N = 41) and French (N = 245) caregivers. The caregivers were primarily mothers, 63.4% for Chilean caregivers and 67.1% for French caregivers (p = 0. 646). Chilean caregivers were younger, and lived more frequently in the same home with the individual with schizophrenia than French caregivers (respectively p = 0.013, and p = 0.001). A majority of caregivers was unemployed (61.5%), but there was no statistically difference between the two groups (p = 0.428).

**Table 1:** Characteristics of Chilean and French caregivers and individuals with schizophrenia

	**Chilean** **(N = 41)**		**French (N = 245)**		**p-value**
	**N**	**(%)**	**N**	**(%)**	
**1. Caregivers**					
Relationship (mother)	26	(63.4)	165	(67.1)	0.646
Age (years), M(SD) §	54.3	(15.1)	60.6	(9.5)	**0.013**
Living situation (with individual with schizophrenia)	37	(90.2)	156	(64.7)	**0.001**
Employment status (employed)	18	(43.9)	92	(37.4)	0.428
SF36+, M(SD)					
Physical functioning	63.4	(24.9)	78.3	(23.4)	**<0.001**
Social functioning	55.8	(22.7)	62.5	(23.9)	0.095
Role physical	45.1	(40.4)	55.9	(40.1)	0.114
Role emotional	43.1	(38.9)	47.7	(41.2)	0.503
Mental health	44.5	(22.0)	51.4	(18.7)	**0.034**
Vitality	38.4	(17.7)	46.4	(19.7)	**0.015**
Bodily pain	51.0	(20.3)	57.2	(25.0)	0.082
General health	49.4	(13.4)	55.5	(21.6)	**0.018**
Mental composite score	35.5	(11.4)	37.4	(10.5)	0.287
Physical composite score	42.8	(8.2)	46.9	(10.0)	**0.013**
**2. Individuals with schizophrenia**					
Gender (male)	26	(63.4)	166	(67.8)	0.584
Age (years), M(SD)	33.2	(8.4)	31.7	(9.1)	0.315

§ M+/−SD: mean +/− standard deviation.

+ range [0–100], 0 lowest and 100 highest level of QoL.

The SF36 scores were globally low in the two groups, especially on the mental scores. Whatever the country, caregivers reported a lower mental QoL level (MCS < 40) than physical QoL level (PCS > 40). There was no statistical significant difference in SF36 mental dimension scores between the two groups, except for MH dimension which was lower in Chilean caregivers. On the contrary, Chilean caregivers reported lower physical SF36 scores than French caregivers (PF, VIT, GH, and PCS).

### Factors associated to caregivers’ QoL of individuals with BD and MD

The results of the univariate and multivariate analyses are presented in Tables 2 and 3. In the multivariate analysis, the relationship with the individual with schizophrenia and the country were the most regular features associating to QoL. Being mother was significantly associated to a lower QoL on all the SF36 dimensions and composite scores, and Chilean caregivers presented lower QoL level for 6 dimensions (PF, SF, RPP, MH, VIT) and PCS. In a lesser extent, living with the patient and being unemployed were associated to lower QoL levels (respectively RPP, REP and MCS; and BP, PCS). On the contrary, no relationship was found with socio-demographic characteristics of individuals with schizophrenia.

**Table 2:** Factors associated with SF36 scores: univariate analyses

		**PFM (SD)^§^ or R^#^**	**SF**	**RPP**	**REP**	**MH**	**VIT**	**BP**	**GH**	**PCS**	**MCS**
1. Caregiver											
Relationship	Mother	73.1(24.8)*	57.3(23.7)**	48.5(40.1)*	42.9(39.5)*	47.5(18.9)**	42.4(19.2)**	53.4(24.7)*	52.5(20.6)*	45.2(9.9)*	35.6(10.2)*
	Other	82.1(21.5)	70.1(21.7)	65.8(38.1)	55.3(42.5)	56.1(18.9)	51.0(19.3)	62.3(23.0)	58.7(20.5)	48.5(9.4)	40.2(10.9)
Age		−0.22**	−0.05	−0.13*	−0.06	0.04	−0.02	−0.15*	−0.15*	−0.23**	0.05
Living situation	with	74.5(24.8)	60.0(24.6)	50.3(40.5)*	44.2(41.0)	48.7(19.2)*	43.6(19.1)*	55.6(25.3)	53.8(21.3)	45.8(10.1)	36.3(10.4)*
	without	79.9(21.7)	65.5(22.1)	63.5(38.8)	54.0(39.8)	54.0(19.5)	48.9(20.3)	58.3(23.0)	56.5(19.1)	47.5(9.1)	39.2(11.0)
Employment status	Yes	82.9(19.2)**	65.0(23.0)	62.2(37.7)*	53.1(40.5)	50.3(18.0)	47.1(18.4)	62.8(23.3)**	60.0(19.1)**	49.6(8.4)**	37.4 (10.5)
	No	71.9(25.8)	59.4(24.1)	49.4(41.1)	43.3(40.7)	50.4(20.1)	44.2(20.3)	52.4(24.3)	51.3(21.0)	44.3(10.1)	37.0(10.8)
2. Individual with schizophrenia											
Gender	Male	77.0(23.1)	60.9(23.3)	51.8(40.8)	44.4(41.5)	49.0(19.1)	44.7(19.3)	56.7(24.3)	54.0(19.6)	46.6(9.5)	36.3(10.6)
	Female	74.0(26.1)	62.9(24.8)	58.9(38.8)	51.8(39.1)	52.8(19.5)	46.2(20.1)	55.2(24.7)	55.5(22.7)	45.6(10.6)	38.9(10.5)
Age		−0.21**	−0.05	−0.12	−0.08	−0.07	−0.07	−0.22**	−0.18*	−0.23**	0.02
3. Country	Chile	63.4(24.9)**	55.8(22.7)	45.1(40.4)	43.1(38.9)	44.5(22.0)*	38.4(17.7)*	51.0(20.3)	49.4(13.4)*	42.8(8.2)*	35.5(11.4)
	France	78.2(23.4)	62.5(23.9)	55.9(40.1)	47.7(41.2)	51.4(18.7)	46.4(19.7)	57.2(25.0)	55.4(21.6)	46.9(9.1)	37.4(10.5)

SF-36—PF: Physical Functioning; SF: Social Functioning; RPP: Role—Physical Problems; REP: Role—Emotional Problems; MH: Mental Health; VIT: Vitality; BP: Bodily Pain; GH: General Health; PCS: Physical Composite Score; MCS: Mental Composite Score.

§ M+/−SD: mean +/− standard deviation; # R : Spearman’s correlation coefficient;* p ≤ 0.05; ** p ≤ 0.01.

**Table 3:** Factors associated with SF36 scores: multivariate analyses

		**PFß^#^**	**SF**	**RPP**	**REP**	**MH**	**VIT**	**BP**	**GH**	**PCS**	**MCS**
1. Caregiver											
Relationship	Mother/Other	−0.20*	−0.26**	−0.21*	−0.17*	−0.23*	−0.21*	−0.20*	−0.16*	−0.17*	−0.22**
Age		−0.23*	−0.11	−0.15	−0.04	−0.02	−0.04	−0.03	−0.04	−0.15	0.05
Living situation	With/without	−0.08	−0.11	−0.19*	−0.15*	−0.11	−0.11	−0.08	−0.07	−0.08	−0.14*
Employment status	Yes/No	0.12	0.09	0.12	0.11	0.02	0.06	0.15*	0.14	0.18*	0.05
2. Individual with schizophrenia											
Gender	Male/Female	−0.03	0.04	0.10	0.07	0.08	0.05	−0.02	0.05	0.01	0.10
Age		−0.02	0.01	−0.03	−0.06	−0.01	−0.02	−0.14	−0.10	−0.06	−0.08
3. Country	Chile/France	−0.27**	−0.14*	−0.11*	−0.04	−0.13*	−0.16*	−0.09	−0.11	−0.18*	−0.07

SF-36—PF: Physical Functioning; SF: Social Functioning; RPP: Role—Physical Problems; REP: Role—Emotional Problems; MH: Mental Health; VIT: Vitality; BP: Bodily Pain; GH: General Health; PCS: Physical Composite Score; MCS: Mental Composite Score.

# ß: standardised beta coefficient (ß represents the change of the standard deviation in QoL score resulting from a change of one standard deviation in the independent variable); * p ≤ 0.05; ** p ≤ 0.01.

## Discussion

To our knowledge, this is the first study to investigate QoL among caregivers of individuals with schizophrenia between a developing and a developed country. There are several important findings in our study.

First, caregivers of individuals with schizophrenia experienced particularly low QoL levels in Chile and France, across multiple domains of QoL including physical and psychological dimensions. This finding confirms previous studies on the significant impact of being a caregiver, in particular in individuals with mental disorders [3]. In France as in other western countries, family has taken functions which were performed in the past by psychiatric institutions [11]. However, the French health system has not sufficiently taking into account this change in the development of health programs and policies. Hospital-based care has still an overwhelming importance, and community services are not sufficiently developed [23]. In Chile, although a communitarian approach was implemented over the last decade [24], public health services approximately cover 80% of the population, psychiatric services are poorly staffed [25], and as a consequence care for patients with schizophrenia is mainly taken by the caregivers [13].

Second, Chilean caregivers reported lower physical SF36 scores than French caregivers. This difference may be explained by a better access to and higher availability of health and economic resources for caregivers in France [26]. In Chile, economic burden may be playing an important role in caregivers’ QoL. Lack of psychiatrist, day hospitals, access to drug treatments, among others, could generate a considerable concern in these relatives [2,25]. Beyond these explanations, it is important to note QoL levels remain low for the physical dimensions in France.

Third, caregivers’ QoL did not statistically differ between Chile and France for mental and psychological QoL dimensions. This finding may appear paradoxical because Chile is characterized by a lower-quantity or quality mental health system than in France. We can hypothesize that emotional and psychological impact is more related to distressing notions such as shame, embarrassment, stigma, rather than burden of care related to household such as taking care of daily tasks. This finding suggests that family interventions should focus on general coping strategies, on the improvement of the social network, on stigma reduction, and on the development of personal strength.

Finally, consistent with prior research [3], the current analysis also find that mothers were generally the primary caregivers and that they reported a lower QoL than other types of informal caregivers. Mothers may experience a higher burden because they are responsible for most aspects of the patients’ daily care [13]. Working life was significantly associated with better QoL in our study. For Magliano and colleagues [27], professional and social network supports represent crucial resources for reducing family burden in schizophrenia. Lastly, the living situation of caregivers and their patients was also an important factor associated with QoL, and our results support the fact that opportunities for residential care should in principal increase family QoL [15].

### Limitations and perspectives

Some limitations have to be carefully considered. The sample may not be representative of the entire population of caregivers, especially in Chile. Confirmation is therefore needed on more diverse and large groups of patients. Moreover, although our models account for a large set of potentially relevant variables, other factors might have increased their explanatory power, such as patients’ characteristics (illness duration, symptom severity…). These factors should be studied in future studies. Finally, our study concerned a generic type of QoL instrument (SF-36). Although SF-36 has good psychometric properties among caregivers of people with mental disorders [22] and is suitable for use across caregivers of individuals with a broad range of mental health problems [28], an important limitation is its relevance when applied to a specific population. Future studies should be proposed using specific questionnaires for caregivers of individuals with schizophrenia [6,29]. Finally, our findings should also be confirmed on objective aspects of caregiving, defined as any disturbance in the family environment due to the patient's disorder and which is potentially verifiable and observable. This analysis could fruitfully compare the findings between the uses of “subjective” or “objective” assessment instruments.

## Conclusion

Despite differences between Chile and France, especially in terms of quality and quantity of mental health services and economic supports, caregivers’ QoL levels remain particularly low for both countries. Our findings suggest that the specific needs of caregivers are not met, whatever the level of performance of the health care systems. Our findings emphasize the need for service providers and policy makers to engage families and service users in policy, planning and practice. There is a failure to use effective clinical approaches in working with families as well as failures in communication in most services worldwide. Future support programmes should address the specific needs of caregivers.

## Competing interest

The Authors have declared that there are no conflicts of interest in relation to the subject of this study.

## Author’s contribution

Both authors contributed equally to this work. All authors read and approved the final manuscript.

## Role of funding source

In France: none.

In Chile, this research was funded by the Universidad de Tarapacá through Proyecto de Investigación Ciencia y Tecnología 3731–12.

## Supplementary Material

Additional file 1 **Chile’s health system is composed of a public health insurance (FONASA) covering about 69 percent of the population, and a private insurance plans (ISAPREs) covering 17 percent of the population.** The remaining population is affiliated with other public agencies (such as Military Health Services) or is without coverage. Public health insurance (FONASA and other public agencies) and public health services approximately cover 80% of the population, and guaranty a relatively equitable access to health care. Concerning care for psychiatric patients, a communitarian approach was implemented over the last decade. Patients may benefit from short-stay in public hospitals in the acute phase, and outpatient care especially by monthly delivering medication by nurses. Care for these patients is mainly taken by their relatives, also known as informal caregivers, who are generally women. However, there is no systematic support for families who rather turn to informal support networks such as church or self-help groups. The French health system combines universal coverage with a public–private mix of hospital and ambulatory care and a higher volume of service provision than in Chile. French public mental health services are organised in “sectors”, each sector catering for a mean population of 54,000 inhabitants. This organisation was aimed at insuring equal access to care whatever the place of residence. In France as in other western countries, psychiatric institutions began discharging mentally ill patients into the community and family has taken functions which were performed in the past by psychiatric institutions. Families of patients suffering from schizophrenia, especially mothers, were more and more involved in the therapeutic process. However, the French health system has not sufficiently taking into account this change in the development of health programs and policies. Hospital-based care has an overwhelming importance, and community services are not sufficiently developed. Click here for file
