# Supplementary material for: Quality of life among caregivers of patients with schizophrenia: a cross-cultural comparison of Chilean and French families
Source: BMC Fam Pract. 2012 May 28;13:42. doi: 10.1186/1471-2296-13-42 (PMC3464874; doi:10.1186/1471-2296-13-42)
Supplement: Additional file 1 — Chile’s health system is composed of a public health insurance (FONASA) covering about 69 percent of the population, and a private insurance plans (ISAPREs) covering 17 percent of the population. The remaining population is affiliated with other public agencies (such as Military Health Services) or is without coverage. Public health insurance (FONASA and other public agencies) and public health services approximately cover 80% of the population, and guaranty a relatively equitable access to health care. Concerning care for psychiatric patients, a communitarian approach was implemented over the last decade. Patients may benefit from short-stay in public hospitals in the acute phase, and outpatient care especially by monthly delivering medication by nurses. Care for these patients is mainly taken by their relatives, also known as informal caregivers, who are generally women. However, there is no systematic support for families who rather turn to informal support networks such as church or self-help groups. The French health system combines universal coverage with a public–private mix of hospital and ambulatory care and a higher volume of service provision than in Chile. French public mental health services are organised in “sectors”, each sector catering for a mean population of 54,000 inhabitants. This organisation was aimed at insuring equal access to care whatever the place of residence. In France as in other western countries, psychiatric institutions began discharging mentally ill patients into the community and family has taken functions which were performed in the past by psychiatric institutions. Families of patients suffering from schizophrenia, especially mothers, were more and more involved in the therapeutic process. However, the French health system has not sufficiently taking into account this change in the development of health programs and policies. Hospital-based care has an overwhelming importance, and community services are not sufficiently develope [file 1471-2296-13-42-S1.doc]

**Additional file 1** Chile’s health system is composed of a public health insurance (FONASA) covering about 69 percent of the population, and a private insurance plans (ISAPREs) covering 17 percent of the population. The remaining population is affiliated with other public agencies (such as Military Health Services) or is without coverage. Public health insurance (FONASA and other public agencies) and public health services approximately cover 80% of the population, and guaranty a relatively equitable access to health care. Concerning care for psychiatric patients, a communitarian approach was implemented over the last decade. Patients may benefit from short-stay in public hospitals in the acute phase, and outpatient care especially by monthly delivering medication by nurses. Care for these patients is mainly taken by their relatives, also known as informal caregivers, who are generally women. However, there is no systematic support for families who rather turn to informal support networks such as church or self-help groups. The French health system combines universal coverage with a public–private mix of hospital and ambulatory care and a higher volume of service provision than in Chile. French public mental health services are organised in “sectors”, each sector catering for a mean population of 54,000 inhabitants. This organisation was aimed at insuring equal access to care whatever the place of residence. In France as in other western countries, psychiatric institutions began discharging mentally ill patients into the community and family has taken functions which were performed in the past by psychiatric institutions. Families of patients suffering from schizophrenia, especially mothers, were more and more involved in the therapeutic process. However, the French health system has not sufficiently taking into account this change in the development of health programs and policies. Hospital-based care has an overwhelming importance, and community services are not sufficiently developed.
